# Evaluation of single-delay arterial spin labeling-based spatial coefficient of variation and histogram-based parameters in relation to cerebrovascular reserve in patients with Moyamoya disease

**DOI:** 10.3389/fneur.2023.1137046

**Published:** 2023-05-30

**Authors:** Markus Fahlström, Teodor Svedung Wettervik, Per Enblad, Anders Lewén, Johan Wikström

**Affiliations:** ^1^Department of Surgical Sciences, Neuroradiology, Uppsala University, Uppsala, Sweden; ^2^Department of Medical Sciences, Neurosurgery, Uppsala University, Uppsala, Sweden

**Keywords:** Moyamoya, arterial spin label (ASL) MRI, spatial coefficient of variation, cerebrovascular reserve, arterial transit time artefact (ATA)

## Abstract

**Introduction:**

Single-delay Arterial Spin Labeling (ASL)-based spatial coefficient of variation (CoV_CBF_) has been suggested as a measure of hemodynamic disturbance in patients with cerebrovascular diseases. However, spatial CoV_CBF_ and other histogram-based parameters such as skewness and kurtosis and the volume of the arterial transit time artefact (ATA_vol_), has not been evaluated in patients with MMD nor against cerebrovascular reserve (CVR). The aim of this study was to assess whether any associations between spatial CoV_CBF_, skewness, kurtosis, and ATA_vol_ are present and to analyze any potential associations with CVR, derived from single-delay ASL in patients with MMD.

**Methods:**

Fifteen MMD patients were included before or after revascularization surgery. Cerebral blood flow (CBF) maps were acquired using pseudo-continuous ASL before, and 5, 15, and 25 min after an intravenous acetazolamide injection. CVR_max_ was defined as the highest percentual increase in CBF at any of the three post-injection time points. A vascular territory template was spatially normalized to each patient, including the bilateral anterior, middle, and posterior cerebral arteries. All affected anterior and middle cerebral artery regions and all unaffected posterior cerebral artery regions were included, based on Suzuki grading by digital subtraction angiography.

**Results:**

Significant differences between affected and unaffected regions were found for CBF, CVR_max_, and ATA_vol_. No association was found between CVR_max_ and any other parameter. High correlations were found between spatial CoV_CBF_, skewness and ATA_vol_.

**Conclusion:**

Spatial CoV_CBF_ derived from single-delay ASL does not correlate with CVR in patients with MMD. Moreover, skewness and kurtosis did not provide additional information of clinical value.

## 1. Introduction

Moyamoya disease (MMD) is a cerebrovascular disease characterized by steno-occlusion at the arteries centered on the terminal portion of the intracranial internal carotid artery (1). The steno-occlusive lesion will cause a progressive decline in cerebral perfusion pressure affecting regional cerebral blood flow (CBF). To compensate, the brain will increase the oxygen extraction fraction (OEF) and dilate resistance arteries to increase CBF and meet the cerebral metabolic demand. Cerebrovascular reserve (CVR) reflects the remaining possible vasodilation and is an important predictor of ischaemic events and prognostic factor for patients with MMD, and can be assessed by measuring CBF before and after administration of acetazolamide (ACZ) (2, 3). In parallel, collaterals from, e.g., the circle of Willis, the ophthalmic artery and leptomeningeal vessels are developed, including the so-called basal Moyamoya vessels (4).

Arterial spin labeling (ASL) is a non-invasive magnetic resonance (MR)-based method to acquire perfusion-weighted data that can be computed into parametric images of CBF with known clinical potential in several cerebral disorders (5). The spatial coefficient of variation (CoV_CBF_) of CBF images (i.e., standard deviation divided by the average CBF for a given region-of-interest) derived from single post-label delay (PLD) ASL acquisition has been evaluated as a measure of hemodynamic disturbance in patients with different cerebrovascular diseases (6–11). This was first proposed by Mutsaerts et al. in 2017 building on the inherent dependency of single PLD ASL on the arterial transit time (ATT) and demonstrated a high correlation between spatial CoV_CBF_ and ATT measured with FEAST (9), a relationship that was further confirmed by Ibaraki et al. (7). When ATT exceeds PLD for a given ASL acquisition, the incomplete arrival of the ASL signal will lead to artefacts characterized by reduced ASL signal in tissue in combination with high intravascular ASL signal (arterial transit time artefact, ATA) (9, 12).

**Figure 1:**
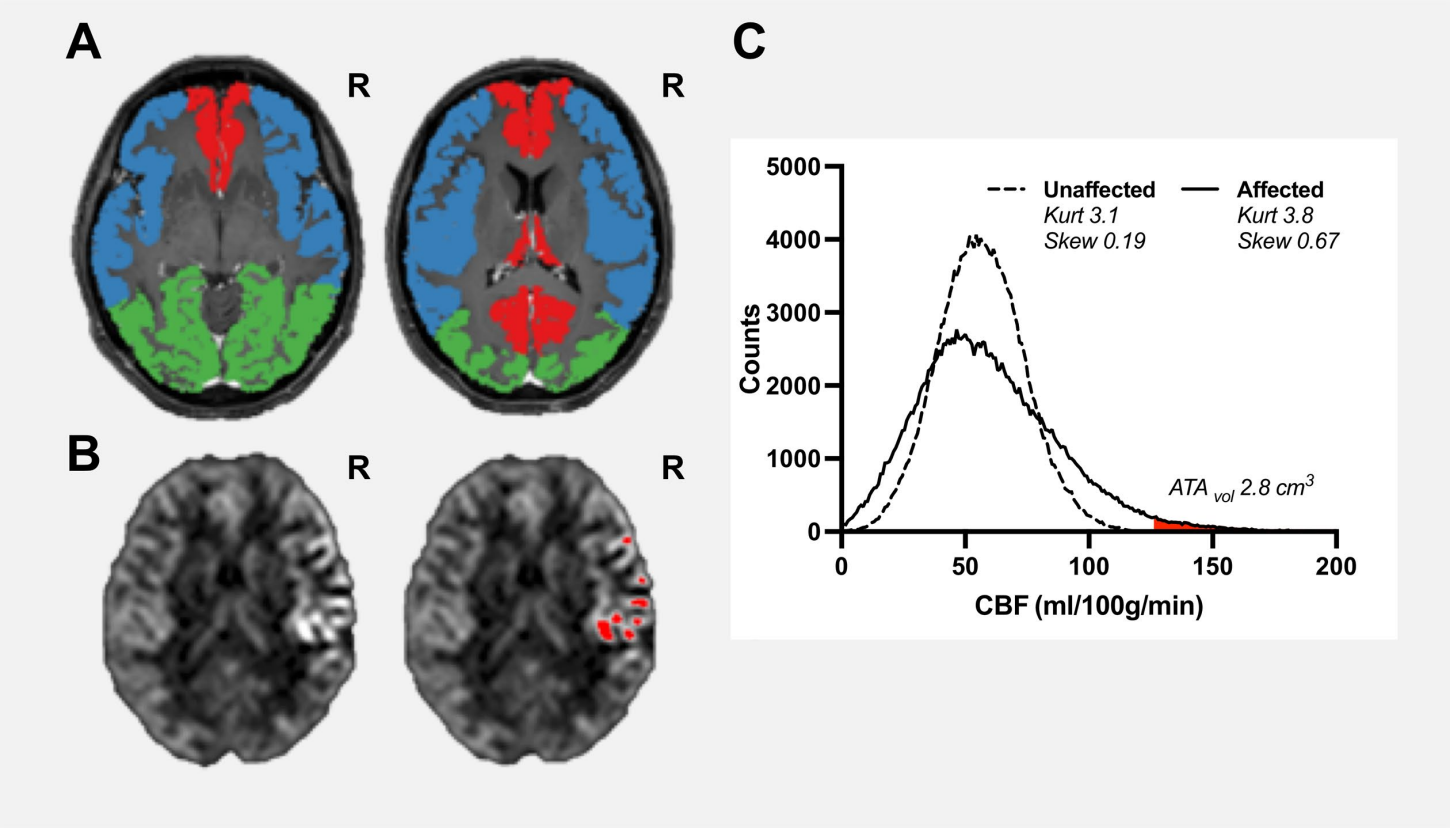
Pre-operative examination of a female patient aged 38  years, with confirmed unilateral Moyamoya disease (Suzuki grade II, right ICA). Vascular regions including bilateral anterior cerebral artery (red), middle cerebral artery (blue), and posterior cerebral artery (green) are shown on a contrast-enhanced T1-weighted image **(A)**. Cerebral blood flow maps **(B)**, slice corresponding to right contrast-enhanced T1-weigheted image in panel **(A)** are shown with clear high intravascular arterial transit time artefacts within the right middle cerebral artery region where red regions-of-interests are pixels identified as arterial high intravascular arterial transit time artefacts using histogram-analysis with a mathematically-defined threshold. ATA_vol_ for the affected vascular region was estimated to 2.8  cm^3^ **(C)**. The histogram of the unaffected region is close to normally distributed (kurtosis 3.1 and skewness 0.19). In comparison, the affected region shows a moderate positive skewness (kurtosis 3.8 and skewness 0.69).

Hence, in a region-of-interest with long ATT, the distribution of CBF values is broadened and consequently the standard deviation of the average CBF in the region-of-interest will increase (see Figure 1). Thus, spatial CoV_CBF_ will be higher in regions with longer ATT compared to regions with normal ATT.

Another approach is to analyse the distribution of CBF values in the vascular territory using histogram analysis. Skewness is a measure of lack of symmetry of a given distribution and is zero for a normally distributed dataset, whereas kurtosis is a measure of whether the distribution is heavy-tailed or light-tailed and is 3 for a normally distributed dataset. Visually and theoretically, it is clear that in regions with long ATT the histogram is positively skewed towards low CBF values with the right tail corresponding to abnormally high CBF values due to intravascular ATA as shown in Figure 1 (7, 13). The presence of intravascular ATA has been used as a visual collateral grading scale in patients with MMD thus indicating clinical potential (12). Furthermore, subregions of intravascular ATAs can be defined by carefully selecting a cut-off value of the right tail and can be quantified as a volume (denoted ATA_vol_) (13). Considering the apparent relationship between spatial CoV_CBF_, ATA_vol_, and histogram-based parameters skewness and kurtosis, these parameters may show further potential as a measure of hemodynamic disturbance in comparison to spatial CoV_CBF_.

In light of the clinical potential of spatial CoV_CBF_, Hara et al. (6) concluded that spatial CoV_CBF_ may help identify increased OEF in affected regions in patients with MMD. Furthermore, associations between increased OEF and decreased CVR have been demonstrated (14, 15). As such, there may exist a relationship between spatial CoV_CBF_ and CVR which, if confirmed, suggests that spatial CoV_CBF_ could replace CVR as a biomarker for hemodynamic disturbance in patients with MMD and no ACZ injection would be necessary.

The aim of this study was to assess any potential associations between spatial CoV_CBF_ and CVR in patients with MMD, and to assess whether histogram-based parameters show equal or higher potential as measures of hemodynamic disturbance in comparison to spatial CoV_CBF_.

## 2. Materials and methods

### 2.1. Patients

Fourteen patients with confirmed bilateral or unilateral MMD were retrospectively included in this study. MRI examinations were performed before and/or after indirect revascularization surgery. The patients were graded using the Suzuki Score system by an experienced neurointerventionist based on digital subtraction or magnetic resonance angiography (MRA) (16). Grading was based on MRA in two patients. Vascular regions of anterior cerebral artery (ACA) and middle cerebral artery (MCA) were included as affected vascular regions if Suzuki grading was higher than 0 and posterior cerebral artery (PCA) was included as unaffected vascular region if Suzuki grading was 0. Hence all unaffected ACA and MCA vascular regions and affected PCA vascular regions were excluded from further analysis. This retrospective study was done in accordance with the declaration of Helsinki and was approved by the Swedish Ethical Review Authority, and all included patients or legal guardians signed an informed consent.

### 2.2. Magnetic resonance imaging

Imaging was performed on a 3.0 T MRI system (dStream Achieva, Philips Healthcare, Best, The Netherlands) using a 32-channel head coil. A 3D pseudo-continuous ASL (pCASL) with background-suppressed gradient spin-echo read-out using a post-label delay (PLD) of 2,500 ms and label duration of 1800 ms was acquired. Acquisition duration was 5 min and 31 s, with a repetition time of 4,735 ms and echo time of 10.7 ms; spatial resolution was 3 × 3 × 6 mm^3^ and number of slices was 14. Acquisition was performed with two background suppression pulses and without flow-crushing gradients. The labeling plane was placed perpendicular to the brain feeding arteries with the aid of a phase contrast MRA survey. The cerebrum was prioritized, leaving the cerebellum not included within the field-of-view. The 3D-pCASL acquisition was performed before (baseline) and repeated 5, 15 and 25 min after ACZ injection (1 g/kg being given to adults and 10 mg/kg to children). Structural 3D T2-weighted fluid attenuated inversion recovery (FLAIR) and 3D contrast enhanced T1-weighted (CE-T1WI) images were acquired with spatial resolution 0.625 × 0.625 × 0.560 and 0.938 × 0.938 × 1 mm^3^ for tissue segmentation and registration purposes, respectively. Of note, the CE-T1WI image was acquired after the 3D-pCASL acquisitions. Furthermore, a 3D time-of-flight MRA (spatial resolution 0.35 × 0.69 × 1.0 mm^3^) was included and used for Suzuki grade assessment if a digital subtraction angiography was missing.

### 2.3. CBF calculation and image processing

The data were analysed using ExploreASL version 1.9.0 (17), running SPM12 toolbox revision 7,771 (Wellcome Trust Center for Neuroimaging, London, United Kingdom), and Matlab version 2022a (Mathworks, MA, United States). In short, CE-T1WI images were segmented into grey matter probability maps (pGM). The deformation field defining the transformation from patient-space to MNI space was derived based on each patient’s CE-T1WI image using SPM12’s Normalise routine. A vascular template defined in MNI space was transformed to patient-specific space using the inverse deformation field including vascular regions of ACA, MCA, and PCA. All vascular regions were up-sampled to match the resolution of the CE-T1WI image applying nearest neighbour interpolation and masked with the corresponding pGM map.

All ASL control/label pairs were corrected for possible motion. Perfusion-weighted and M0 images were then rigid-body registered to the patient’s pGM map. Quantification of CBF was performed using a single compartment model (5). Partial volume correction was performed by the method described by Asllani et al. (18). CBF images were up-sampled to match the resolution of the CE-T1WI image applying trilinear interpolation.

An in-house developed Matlab script imported CBF images and extracted masked vascular regional values of mean CBF, standard deviation (SD), kurtosis and skewness and calculated CVR (as defined by Eq. (1) for 5 min, 15 min, and 25 min post-ACZ CBF measurements, respectively) and spatial CoV_CBF_ (as defined by Eq. (2)).


(1) $$\mathrm{CVR}=\frac{{\mathrm{CBF}}_{\mathrm{post}-\mathrm{ACZ}}-{\mathrm{CBF}}_{\mathrm{baseline}}}{{\mathrm{CBF}}_{\mathrm{baseline}}}$$

(2) $$\mathrm{spatial}\;\;{\mathrm{CoV}}_{\mathrm{CBF}}=\frac{\sigma \left({\mathrm{CBF}}_{\mathrm{Region}}\right)}{\mu \left({\mathrm{CBF}}_{\mathrm{Region}}\right)}\times 100\%$$

The maximum achieved CVR (CVR_max_) post-injection was used in the subsequent data analysis. The volume of regional high intravascular signal (ATA_vol_) was defined as the right tail of the regional histogram with a mathematically given cut-off. The cut-off was calculated as median CBF + 3 times the median absolute difference (17). The volume, given in cm^3^, was calculated as the total number of pixels above the cut-off multiplied by the CBF image resolution (see Figure 1). Spatial occurrence of ATA pixels was inspected visually to ensure enclosed within high intravascular signal in the CBF images.

### 2.4. Statistical analysis

Derived parameters included in the statistical analysis were CBF, CVR_max_, CoV_CBF_, skewness, kurtosis, and ATA_vol_ further divided into regions of ACA, MCA or PCA. Descriptive analysis was performed using means and SD. The D’Agostino-Pearson normality test (omnibus K2) was used to test each parameter within each vascular region for normality.

A correlation matrix for each vascular region was derived using Spearman’s rho including all parameters to test whether any association were present. A repeated-measures one-way analysis of variance (ANOVA) with Tukey’s multiple comparisons test was performed to compare all vascular regions for each parameter separately. Derived *p*-values are two-sided and presented as exact values or <0.01 if below 0.01, where *p* < 0.05 is considered statistically significant. GraphPad Prism 9 for Mac (GraphPad Software, La Jolla, CA, United States) was used for statistical analysis and graph design.

## 3. Results

The median age of the 14 included patients at MRI acquisition was 29 years (range 10 to 53 years). Eleven patients had bilateral disease and three patients had unilateral disease (Suzuki grade 0 on contralateral side). Eight patients had undergone previous revascularization surgery (indirect/direct – 7/1). Table 1 summarizes the clinical and demographic information for the patients studied.

**Table 1:** Patient characteristics.

#	Age*/Sex	Side	Suzuki grade	Operation
1	19/M	L	II	MBH L
2	29/F	L/R	I/V	MBH R
3	31/F	L/R	III	MBH L/R
4	38/F	R	II	
5	53/F	L/R	IV	MBH L/R
6	10/F	L/R	III	MBH L/R
7	29/F	L/R	IV/III	MBH L/R
8	24/F	L/R	IV^†^	Bypass/ MBH R
9	52/F	L/R	III	
10	19/F	L/R	IV	
11	50/F	R	III	
12	14/F	L/R	IV	MBH L
13	25/M	L/R	III	
14	14/F	L/R	III^†^	

*Age at first examination. †Suzuki grade assessment by magnetic resonance angiography. F, female; L, left; M, male; MBH, multiple burr holes; R, right.

**Figure 2:**
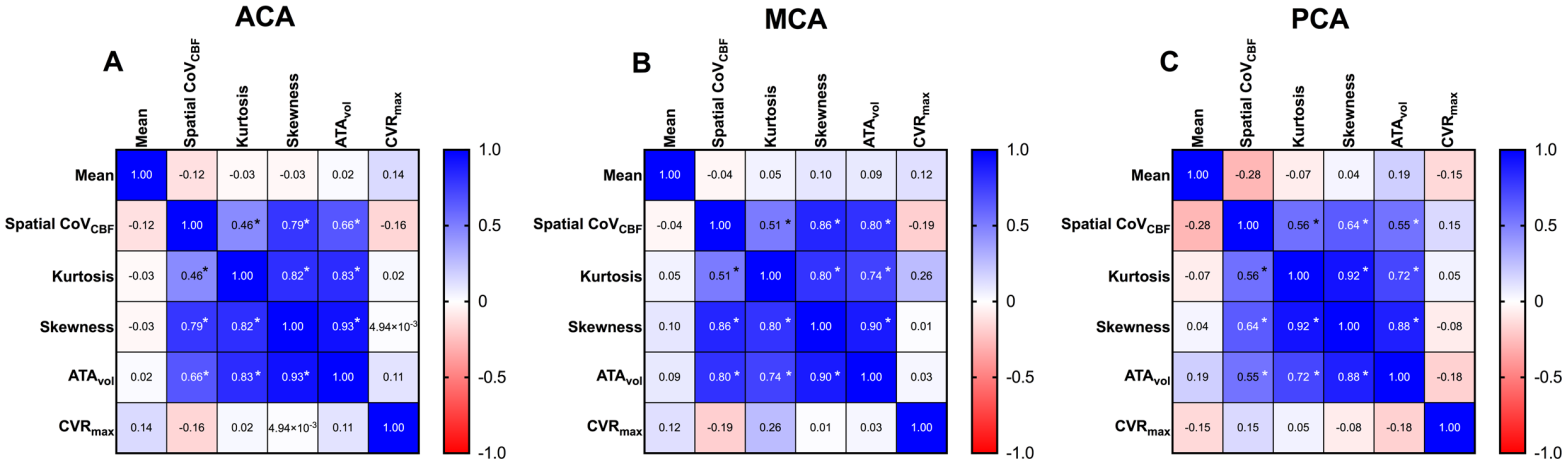
Correlation matrices for anterior cerebral artery (ACA) **(A)**, middle cerebral artery (MCA) **(B)** and posterior cerebral artery (PCA) **(C)** respectively, based on Spearman’s rho.

Mean and SD together with matrices with Spearman’s rho calculated between all derived parameters for all vascular regions are summarized in Table 2 and Figure 2 respectively. CVR_max_ did not demonstrate any correlation with any studied parameter (*r* < 0.3, *p* > 0.05). High correlations were found between spatial CoV_CBF_ and skewness and ATA_vol_ in MCA and ACA regions (*r* = 0.71 to 0.81, *p* < 0.05). Low correlation was found between spatial CoV_CBF_ and kurtosis (*r* = 0.47 to 0.51, *p* < 0.05). Furthermore, ATA_vol_ showed very high correlation with skewness (*r* = 0.92 to 0.94, *p* < 0.05) and high correlation with kurtosis (*r* = 0.78 to 0.84, *p* < 0.05). Skewness was in general positive, indicating right-skewed histograms.

**Table 2:** Mean and standard deviation for all parameters included in the data analysis for each vascular region, respectively.

Parameter	ACA	MCA	PCA
CBF [ml/100 g/min]	45 (9)	46 (11)	37 (10)
CVR_max_ [%]	49 (16)	47 (17)	58 (23)
Spatial CoV_CBF_ [%]	42 (10)	43 (11)	40 (9)
Skewness [a.u]	0.47 (0.35)	0.50 (0.33)	0.45 (0.43)
Kurtosis [a.u]	3.7 (0.9)	3.8 (0.5)	3.8 (1.1)
ATA_vol_ [cm^3^]	0.94 (0.47)	2.20 (0.87)	0.53 (0.28)

ACA, anterior cerebral artery; ATA, arterial transit time artefact; a.u, arbitrary units; CBF, cerebral blood flow; CoV, coefficient of variation; CVR, cerebrovascular reserve; MCA, middle cerebral artery; PCA, posterior cerebral artery.

**Figure 3:**
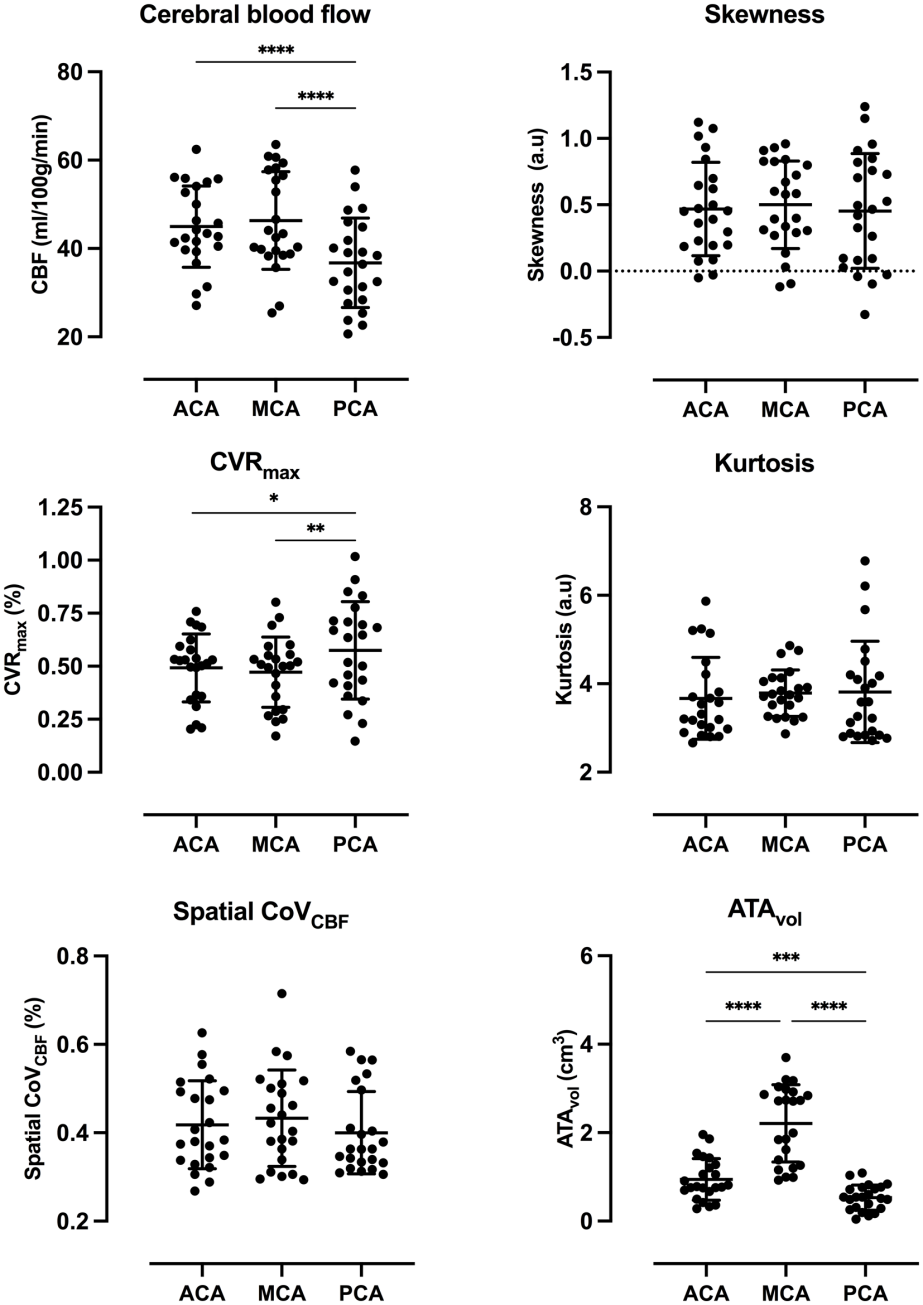
Scatter dot plots for all parameters included in the statistical analysis with average and standard deviation for each vascular region. *p*-value style: <0.05 (^*^), <0.01 (^**^), <0.001 (^***^), and <0.0001 (^****^). Exact *p*-values are presented in Supplementary Table S4.

CBF was significantly lower in PCA compared to ACA and MCA. CVR_max_ was significantly higher in PCA compared to MCA. The same trend was present for ACA vs. PCA and MCA vs. ACA, but did not meet the criteria for statistical significance. ATA_vol_ was significantly higher in MCA compared to PCA and ACA. Significant differences were found between all vascular regions for ATA_vol_. No statistically significant differences were found between any vascular regions for the remaining parameters included in the analysis. Scatter dot plots for all parameters included in the statistical analysis are presented in Figure 3.

## 4. Discussion

The current study demonstrates that associations between CVR and spatial CoV_CBF_, histogram-based parameters and ATA_vol_ are negligible in vascular regions in patients with MMD. Moreover, CBF, CVR_max_, and ATA_vol_ demonstrated significant differences between affected and unaffected vascular regions.

Poorer CVR is expected in affected regions compared to unaffected regions. The difference was higher between MCA and PCA, compared to between ACA and PCA, which can be attributed to MCA being the more affected territory by steno-occlusions.

Ibaraki et al. (7) and Mutsaerts et al. (8) found significant differences in spatial CoV_CBF_ between contralateral and ipsilateral MCA/ICA regions in patients with unilateral steno-occlusive disorder. Only two out of 15 included patients in the present study had unilateral disease, and hence, a similar comparison was not performed because of few data points. However, we did not find any significant difference between affected (MCA or ACA) and unaffected regions (PCA). Of note, the posterior circulation is known to be slower than the anterior, (19) which in theory could add to the heterogenization of the ASL tracer arrival in PCA regions, thus contributing to a higher spatial CoV_CBF_ compared to healthy MCA/ICA regions. Still, we used a PLD of 2,500 ms, which should reduce this effect as shown by Hara et al. (6). Neither Tortora et al. (10) and Hara et al. (6) included spatial CoV_CBF_ from unaffected regions or similar in their analysis of patients with MMD, making comparisons to the present study difficult.

Large differences between ATA_vol_ in affected and unaffected regions were found. ATA_vol_ was not zero within PCA which can be attributed to a slower posterior CBF circulation, i.e., prolonged ATT which can contribute to pixels being defined as intravascular ATAs. Of note, stratification between affected and unaffected regions was easy. Further studies into presence and location of ATA_vol_ are warranted to further investigate possible clinical implications. The calculated values of skewness are in general positive – indicating a right skewed histogram with the tail higher at higher CBF values which is in line with the demonstrated association between skewness and ATA_vol_ (see Figure 1).

Few studies have included spatial CoV_CBF_ on patients with MMD. Tortora et al. (10) concluded that spatial CoV_CBF_ may contribute to predict surgical outcomes in paediatric patients with MMD by demonstrating a significantly decreasing spatial CoV_CBF_ 6, 12, and 24 months after encephalo-duro-arterio-myo-synangiosis compared to baseline. Likewise, Liang et al. (11) reported significantly decreasing spatial CoV_CBF_ after MCA-superficial temporal artery anastomosis in adult MMD patients. Tortora et al. (10) found no changes in average CBF pointing towards the standard deviation of CBF being the driving mechanism, which is further verified by the significant decrease in time to peak calculated from a dynamic susceptibility contrast MRI acquisition, thus indicating a homogenization in ASL tracer arrival. On the contrary, Liang et al. (11) found significantly increasing average CBF values in several sub-divisions of the MCA, but not in the total MCA. Between Tortora et al. (10) and Liang et al. (11) there are several methodological differences presents; pediatric vs. adult patient populations, different revascularization procedures, the ASL acquisitions are performed with different PLDs and CBF values are extracted from different regions-of-interests. Hence, any comparisons should be done with caution and the possible clinical implications of using spatial CoV_CBF_ for post-operative assessment should be further investigated. The current study was not designed for longitudinal assessment and this is therefore beyond the scope of this study.

Hara et al. (6) found that spatial CoV_CBF_ may help identify increased OEF in patients with MMD by comparing ASL-based spatial CoV_CBF_ with OEF. However, this finding is not supported by a study by Ibaraki et al. (7) on patients with steno-occlusion, both estimating OEF from ^15^O-gas PET. ^15^O-gas PET data was not acquired simultaneously in either study, which may compromise comparisons between PET and ASL data (20). Moreover, Ibaraki et al. used a PLD of 2000 ms whereas Hara et al. (6) acquired ASL data using a PLD of 1,525 ms and 2,525 ms, respectively. In the latter case, a PLD of 1,525 ms was found to have better diagnostic capabilities. Differences in how regions-of-interest are defined are also present, which may affect the comparison between the studies. Although, there are differences in the applied methodology between both studies, other explanations may also be put forward.

Spatial CoV_CBF_, and histogram-based parameters are related to delayed CBF (increased ATT) through collateral pathways (8, 9, 12, 21). Furthermore, the presence of collateral pathways has been found to be associated with reduced CVR and increased OEF (22–25). Hence, there may be an indirect link between spatial CoV_CBF_ and OEF and CVR owing to differences in collateral pathways. Thus, correlations between spatial CoV_CBF_ and OEF and CVR could depend on the collaterals present, which may explain the ambiguous results reported by Hara et al. (6) and Ibaraki et al. (7). In the current study we investigated whether CVR was associated with spatial CoV_CBF_ and concluded no relationship. However, Ibaraki et al. (7), on the other hand, found a significant correlation between spatial CoV_CBF_ and CVR (*r* = −0.373, *p* = 0.030); however, CVR measurement was performed using induced hypercapnia and the correlation coefficient was calculated on pooled data (contralateral and ipsilateral values). Although, several studies support the correlation between spatial CoV and ATT, extrapolating this known relationship to other measures of hemodynamic compromise should be done with caution.

### 4.1. Limitations

Included patients are both adults and children with bilateral or unilateral MMD who have or have not undergone revascularization surgery; as such the patients represent a heterogeneous population which may or may not affect the present results. Reports have shown that ATT decreases around 10% during vasodilation secondary to ACZ injection, which can affect CBF quantification using a single-delay PLD ASL acquisition (26, 27). CVR values in the current study may thus be underestimated. Moreover, it has been shown that different PLD could yield different spatial CoV_CBF_ (6). In the present study, the 3D pCASL was acquired with a PLD of 2,500 ms, hence any comparisons made with other reports using different PLD values should be done with caution.

## 5. Conclusion

Regional spatial CoV_CBF_ derived from single-delay pCASL imaging does not correlate with CVR in patients with MMD. Moreover, histogram-based parameters did not provide additional information of clinical value.

## Data availability statement

The raw data supporting the conclusions of this article will be made available by the authors, without undue reservation.

## Ethics statement

This study, involving human participants were reviewed and approved by the Swedish Ethical Review Authority. Written informed consent to participate in this study was provided by all participants or legal guardian.

## Author contributions

MF performed data post-processing, statistical analysis, and drafting the manuscript. MF, TSW, PE, AL, and JW contributed to the conception and design of the study and participated in the data collection and writing process. All authors contributed to the article and approved the submitted version.

## Funding

This project was partly funded by grants from the Erik, Karin, and Gösta Selanders Stiftelse and the Swedish Stroke Association. The funders had no role in study design, data collection, analysis, decision to publish or preparation of the manuscript.

## Conflict of interest

The authors declare that the research was conducted in the absence of any commercial or financial relationships that could be construed as a potential conflict of interest.

## Publisher’s note

All claims expressed in this article are solely those of the authors and do not necessarily represent those of their affiliated organizations, or those of the publisher, the editors and the reviewers. Any product that may be evaluated in this article, or claim that may be made by its manufacturer, is not guaranteed or endorsed by the publisher.
