# Supplementary material for: Evaluation of single-delay arterial spin labeling-based spatial coefficient of variation and histogram-based parameters in relation to cerebrovascular reserve in patients with Moyamoya disease
Source: Front Neurol. 2023 May 30;14:1137046. doi: 10.3389/fneur.2023.1137046 (PMC10261981; doi:10.3389/fneur.2023.1137046)
Supplement: Supplementary file 1 [file Data_Sheet_1.docx]

Supplementary Material

**Table 1.** Correlation matrix with derived Spearmen’s rho and p-values (italic) for anterior cerebral artery. Significant p-values are highlighted in bold.

|  | **Mean** | **Spatial CoV_CBF_** | **Kurtosis** | **Skewness** | **ATT_Vol_** | **CVR_Max_** |
| --- | --- | --- | --- | --- | --- | --- |
| **Mean** | 1.00  *-* | -0.12  *0.58* | 0.03  *0.89* | -0.04  *0.88* | 0.02  *0.94* | 0.14  *0.52* |
| **Spatial CoV_CBF_** | -0.12  *0.58* | 1.00  - | 0.46  ***0.03*** | 0.79  ***<0.01*** | 0.66  ***<0.01*** | -0.16  *0.46* |
| **Kurtosis** | -0.03  *0.89* | 0.46  ***0.03*** | 1.00  - | 0.82  ***<0.01*** | 0.83  ***<0.01*** | 0.02  *0.91* |
| **Skewness** | -0.04  *0.88* | 0.79  ***<0.01*** | 0.82  ***<0.01*** | 1.00  - | 0.93  ***<0.01*** | 0.01  *0.98* |
| **ATT_Vol_** | 0.02  *0.94* | 0.66  ***<0.01*** | 0.83  ***<0.01*** | 0.93  ***<0.01*** | 1.00  - | 0.11  *0.63* |
| **CVR_Max_** | 0.14  *0.52* | -0.16  *0.46* | 0.02  *0.91* | 0.01  *0.98* | 0.11  *0.63* | 1.00  - |

ATA, arterial transit time artefact; CBF, cerebral blood flow; CVR, cerebrovascular reserve; CoV, coefficient of variation

**Table 2.** Correlation matrix with derived Spearmen’s rho and p-values (italic) for middle cerebral artery. Significant p-values are highlighted in bold.

|  | **Mean** | **Spatial CoV_CBF_** | **Kurtosis** | **Skewness** | **ATT_Vol_** | **CVR_Max_** |
| --- | --- | --- | --- | --- | --- | --- |
| **Mean** | 1.00  *-* | -0.04  *0.86* | 0.05  *0.82* | 0.10  *0.65* | 0.09  *0.69* | 0.12  *0.58* |
| **Spatial CoV_CBF_** | -0.04  *0.86* | 1.00  - | 0.52  ***0.01*** | 0.86  ***<0.01*** | 0.80  ***<0.01*** | -0.19  *0.37* |
| **Kurtosis** | 0.05  *0.82* | 0.52  ***0.01*** | 1.00  - | 0.80  ***<0.01*** | 0.74  ***<0.01*** | 0.26  *0.22* |
| **Skewness** | 0.10  *0.65* | 0.86  ***<0.01*** | 0.80  ***<0.01*** | 1.00  - | 0.90  ***<0.01*** | 0.01  *0.96* |
| **ATT_Vol_** | 0.09  *0.69* | 0.80  ***<0.01*** | 0.74  ***<0.01*** | 0.90  ***<0.01*** | 1.00  - | 0.04  *0.87* |
| **CVR_Max_** | 0.12  *0.58* | -0.19  *0.37* | 0.26  *0.22* | 0.01  *0.96* | 0.04  *0.87* | 1.00  - |

ATA, arterial transit time artefact; CBF, cerebral blood flow; CVR, cerebrovascular reserve; CoV, coefficient of variation

**Table 3.** Correlation matrix with derived Spearmen’s rho and p-values (italic) for posterior cerebral artery. Significant p-values are highlighted in bold.

|  | **Mean** | **Spatial CoV_CBF_** | **Kurtosis** | **Skewness** | **ATT_Vol_** | **CVR_Max_** |
| --- | --- | --- | --- | --- | --- | --- |
| **Mean** | 1.00  *-* | -0.28  *0.14* | -0.07  *0.72* | 0.04  *0.85* | 0.19  *0.34* | -0.15  *0.45* |
| **Spatial CoV_CBF_** | -0.28  *0.14* | 1.00  - | 0.56  ***<0.01*** | 0.64  ***<0.01*** | 0.55  ***<0.01*** | 0.15  *0.46* |
| **Kurtosis** | -0.07  *0.72* | 0.56  ***<0.01*** | 1.00  - | 0.92  ***<0.01*** | 0.72  ***<0.01*** | 0.05  *0.80* |
| **Skewness** | 0.04  *0.85* | 0.64  ***<0.01*** | 0.92  ***<0.01*** | 1.00  - | 0.88  ***<0.01*** | -0.08  *0.70* |
| **ATT_Vol_** | 0.19  *0.34* | 0.55  ***<0.01*** | 0.72  ***<0.01*** | 0.88  ***<0.01*** | 1.00  - | -0.18  *0.36* |
| **CVR_Max_** | -0.15  *0.45* | 0.15  *0.46* | 0.05  *0.80* | -0.08  *0.70* | -0.18  *0.36* | 1.00  - |

ATA, arterial transit time artefact; CBF, cerebral blood flow; CVR, cerebrovascular reserve; CoV, coefficient of variation

**Table 4.** Repeated-measures one-way analysis of variance (ANOVA) with Tukey’s multiple comparisons test. Scatter plots of the datasets can be found in Figure 3.

| **Parameter** | **Comparisons** | **Mean Diff** | **95% CI of Diff** | **Adj. p-value** |
| --- | --- | --- | --- | --- |
| **CBF [ml/100g/min]** | **ACA vs MCA**  **ACA vs PCA**  **MCA vs PCA** | -1  8  10 | -3 to 0.8  5 to 11  6 to 13 | 0.26  <0.0001  <0.0001 |
| **CVR_max_ [%]** | **ACA vs MCA**  **ACA vs PCA**  **MCA vs PCA** | 0.020  -0.083  -0.103 | -0.008 to 0.0481  -0.153 to -0.0127  -0.172 to -0.0336 | 0.21  0.02  0.03 |
| **Spatial CoV_CBF_ [%]** | **ACA vs MCA**  **ACA vs PCA**  **MCA vs PCA** | -0.015  0.018  0.033 | -0.059 to 0.029  -0.010 to 0.046  -0.015 to 0.080 | 0.68  0.27  0.22 |
| **Skewness [a.u]** | **ACA vs MCA**  **ACA vs PCA**  **MCA vs PCA** | -0.032  0.015  0.047 | -0.203 to 0.139  -0.152 to 0.182  -0.123 to 0.217 | 0.88  0.97  0.77 |
| **Kurtosis [a.u]** | **ACA vs MCA**  **ACA vs PCA**  **MCA vs PCA** | -0.117  -0.143  -0.027 | -0.662 to 0.429  -0.599 to 0.312  -0.649 to 0.595 | 0.85  0.71  0.99 |
| **ATA_vol_ [cm^3^]** | **ACA vs MCA**  **ACA vs PCA**  **MCA vs PCA** | -1.270  0.408  1.678 | -1.681 to -0.859  0.167 to 0.649  1.301 to 2.055 | <0.0001  0.0009  <0.0001 |

ACA, anterior cerebral artery; ATA, arterial transit time artefact; a.u, arbitrary units; CBF, cerebral blood flow; CoV, Coefficient of Variation; CVR, cerebrovascular reserve; MCA, middle cerebral artery; PCA, posterior cerebral artery
